# Children’s Use of and Experiences With a Web-Based Perioperative Preparation Program: Directed Content Analysis

**DOI:** 10.2196/13565

**Published:** 2019-04-12

**Authors:** Gunilla Lööf, Nina Andersson-Papadogiannakis, Charlotte Silén

**Affiliations:** 1 Department of Learning, Informatics, Management and Ethics Karolinska Institutet Stockholm Sweden; 2 Department of Paediatric Anaesthesia and Intensive Care Karolinska University Hospital Stockholm Sweden; 3 Department of Women's and Children's Health Karolinska Institutet Stockholm Sweden

**Keywords:** child, parents, learning, education, internet, information, preparation, preoperative, anesthesia, risk, delivery of health care

## Abstract

**Background:**

Web-based technology is useful as an alternative means of providing preparation programs to children in pediatric care. To take full advantage of Web-based technology, there is a need to understand how children use and learn from such programs.

**Objective:**

The objective of this study was to analyze children’s use of and experiences with a Web-based perioperative preparation program in relation to an educational framework of children’s learning.

**Methods:**

This study is the final part of a three-phase study in which all families with children aged 3 to 16 years (N=32) admitted for outpatient surgery over 1 week were asked to participate. Children were interviewed before (phase 1) and after (phase 2) anesthesia and surgery and 1 month after hospitalization (phase 3). The data in this study (phase 3) relate to six children (5 to 13 years) who participated in the follow-up interviews in their homes a month after hospitalization. The study used a directed qualitative interpretative approach. The interviews were conducted in a semistructured manner as the children—without guidance or influence from the interviewer—visited and navigated the actual website. The data were analyzed based on a combination of the transcribed interviews and field notes, and were subjected to a previous theoretical investigation based on children’s learning on a website in pediatric care.

**Results:**

Six children, five boys (5-12 years) and one girl (13 years), participated in the follow-up study in their homes a month after hospitalization. The children were selected from the 22 initially interviewed (in phases 1 and 2) to represent a variation of ages and perioperative experiences. The children’s use of and experiences with the website could be explained by the predetermined educational themes (in charge of my learning, discover and play, recognize and identify, and getting feedback), but additional aspects associated with children’s need for identification, recognition, and feedback were also revealed. The children used the website to get feedback on their own experiences and to interact with and learn from other children.

**Conclusions:**

This analysis of children’s use of and experiences with a Web-based preparation program emphasizes the importance of including a theoretical educational framework of children’s learning in the development and design of websites in pediatric care. Creating opportunities for Web-based communication with others facing similar experiences and possibilities for receiving feedback from adults are important factors for future consideration.

## Introduction

### Background

Millions of children around the world are hospitalized every year for anesthesia and surgery. Many experience preoperative anxiety that negatively affects the individual experience, medical outcome, and relations with health care services in both the short and long term [1-5]. Understanding the perioperative procedures and continuous information and interaction with health care providers decreases distress, increases compliance with medical procedures, improves cooperation with health care providers, and reduces postoperative trauma [6-8]. Despite strong evidence that preparation of children for anesthesia and surgery reduces fear and anxiety [7,9,10], the content, design, and availability of preparation programs vary greatly [10,11]. Many children continue to report a lack of information and are forced to experience the perioperative processes unprepared [12,13]. Web-based technology represents a rapidly expanding alternative with almost unlimited opportunities for development and distribution of preparation programs for children in pediatric care [14-16]. Websites should not just provide information but must also incorporate features that allow for children’s need to process information appropriately to understand the content [17-19].

### Learning Concepts

The use of Web-based technology as a tool for children’s preparation and comprehension of perioperative procedures requires knowledge of how children of different ages learn.

In this study, the learning processes are active construction processes in which the individual’s lifeworld forms the basis for their understanding, thinking, and actions [17,19]. Learning involves the whole person using different kinds of information and interactions with people, artifacts, and the environment [19,20]. No one can passively receive understanding and skills from others. Learning is a multifaceted phenomenon that requires processing of information cognitively, emotionally, and socially through testing and practical actions [21,22]. The learning concepts of preunderstanding, motivation, learning processes, and learning outcomes, which were previously used in a theoretical analysis of a Web-based preparation program for children [18], are used in this study to analyze and describe children’s approach to a website consisting of a comprehensive, interactive, age-differentiated multimedia Web-based portal aimed to prepare and educate children and their families before pediatric perioperative care (see Multimedia Appendix 1 for further explanation of central learning concepts).

A new learning situation is understood and interpreted based on the learner’s preunderstanding, knowledge, experiences, attitudes, and behaviors that the individual has internalized over time. Preunderstanding supports understanding of new things but can also become a hindrance to learning [19,21,23]. Misunderstandings and reactions to something that seems odd, different, or frightening can obstruct the will to consider and learn about the unknown [17]. Motivation is vital to stimulate the start and maintenance of a learning process [17,23-25] and can be triggered by curiosity and experiences of something being fun [26]. Another driving force is when previous approaches are not working, and new questions that need to be answered and investigated arise [17,23-25]. Motivation is stimulated both by the challenge to master something, as well as by the feeling of succeeding [27]. The essence of the learning process constitutes the individual’s processing of information by noticing different data through all senses: by listening, watching, touching, smelling, and tasting. A learner not only receives information, but also reflects, tries out, and relates to previous knowledge and thereby constructs new understanding and abilities [21,22]. Learning processes are meant to result in learning outcomes such as understanding, ability to perform skills, and possibly changed attitudes and behavior depending on the learning situation [17,19,28]. In this case, the learning outcomes are related to children and parents being prepared for hospitalization and, specifically, for anesthesia and surgery. The aim is for the child to understand what is going to happen and be able to cope with the situation. Moreover, it is important that both children and parents experience safety and confidence. Feedback on the learning achievements is important to support the learner’s confidence that the message has been understood correctly or to show that the information should be repeated to improve understanding [29,30]. (See Multimedia Appendix 1 for further explanation of central learning concepts.)

### Web-Based Technology in Pediatric Care

The age at which children start using the internet is notably earlier nowadays (67% of 2-year-olds) and the proportion who use it daily increases with age (32% at age 2 years, 50% at age 6 years, 75% at age 10 years, and 96% of teenagers) [31]. Web-based information opens up opportunities for reaching children in their natural venues, from their perspectives, and engaging them as part of their own care. Compared to “traditional education materials” Web-based technology can expand the range of things that children can create and in doing so enable them to encounter ideas that were previously not accessible to them [32]. Web-based technology also enables contact with experts and others facing similar health challenges. As a learning tool, Web-based technology allows tailoring of information for individual needs, a private learning environment, and an immediate reinforcement of the learning that has occurred [32-36]. To take advantage of Web-based technology as a learning resource for children in pediatric care, there is a need to understand how children use and learn from Web-based preparation programs. A previous theoretical analysis of a Web-based preparation program related to the central learning concepts of preunderstanding, motivation, learning processes, and learning outcome resulted in four themes (in charge of my learning, discover and play, recognize and identify, and getting feedback) important for children’s learning with Web-based technology prior to contact with pediatric care [18].

### Objective

The objective of this study was to analyze children’s use of and experiences with a Web-based perioperative preparation program in relation to an educational framework of children’s learning.

## Methods

### Research Approach

A directed qualitative content analysis primarily guided by Hsieh and Shannon [37] was applied to illuminate and explain children’s use of and experiences with a Web-based perioperative preparation program. The chosen approach of content analysis applies predetermined variables or concepts to interpret data and is used when existing theoretical and/or empirical knowledge about a subject is judged to enhance the understanding of a certain research question. The aim is to describe common themes characterizing the object being studied. The directed content analysis approach in this study builds on a previous investigation [18] that provided a theoretical basis of children’s learning on a website. This approach is judged to be appropriate since the analysis is operationalized based on previous knowledge that would benefit from further refinement or development. To further enhance the understanding of how children learn on a website, the themes from the previous study [18] formed the basis for the directed analysis [18,37,38] in this study. This study comprises descriptions of the manifest, concrete content focused on the visible, obvious components and what the children expressed, as well as interpretations of the meaning of what was said, the latent messages, yet still close to the children’s experiences [39,40].

### Data Collection

This study is the final part of a three-phase study in which all families with children aged 3 to 16 years (N=32) admitted for outpatient surgery at Astrid Lindgren Children’s Hospital in Stockholm, Sweden, during 5 days in February 2015, were asked to participate in interviews on their arrival to the hospital. The request was followed by age-differentiated verbal and written information. The families had visited the preoperative assessment clinic, received information from an anesthesiologist, and were advised to use both written brochure information and the Anaesthesia-Web, the website investigated in this study, a month before admittance. Children were interviewed before (phase 1) and after (phase 2) anesthesia and surgery and again at home 1 month after hospitalization (phase 3).

Both children and parents were informed that consent was negotiable, that it was possible to withdraw at any point, and they were guaranteed that their participation would not affect their care or treatment in any way. Prior to the interviews in the third phase, children and parents received additional verbal and written information and renewed consent was obtained by using forms designed and adapted for children with different cognitive abilities. The first author (GL), who is a nurse specialized in anesthesia and pediatrics with extensive experience with talking with children in the health care context, performed the interviews in all different phases. The study was approved by the Regional Ethical Review Board in Stockholm (2014/309-31/3).

**Figure 1:**
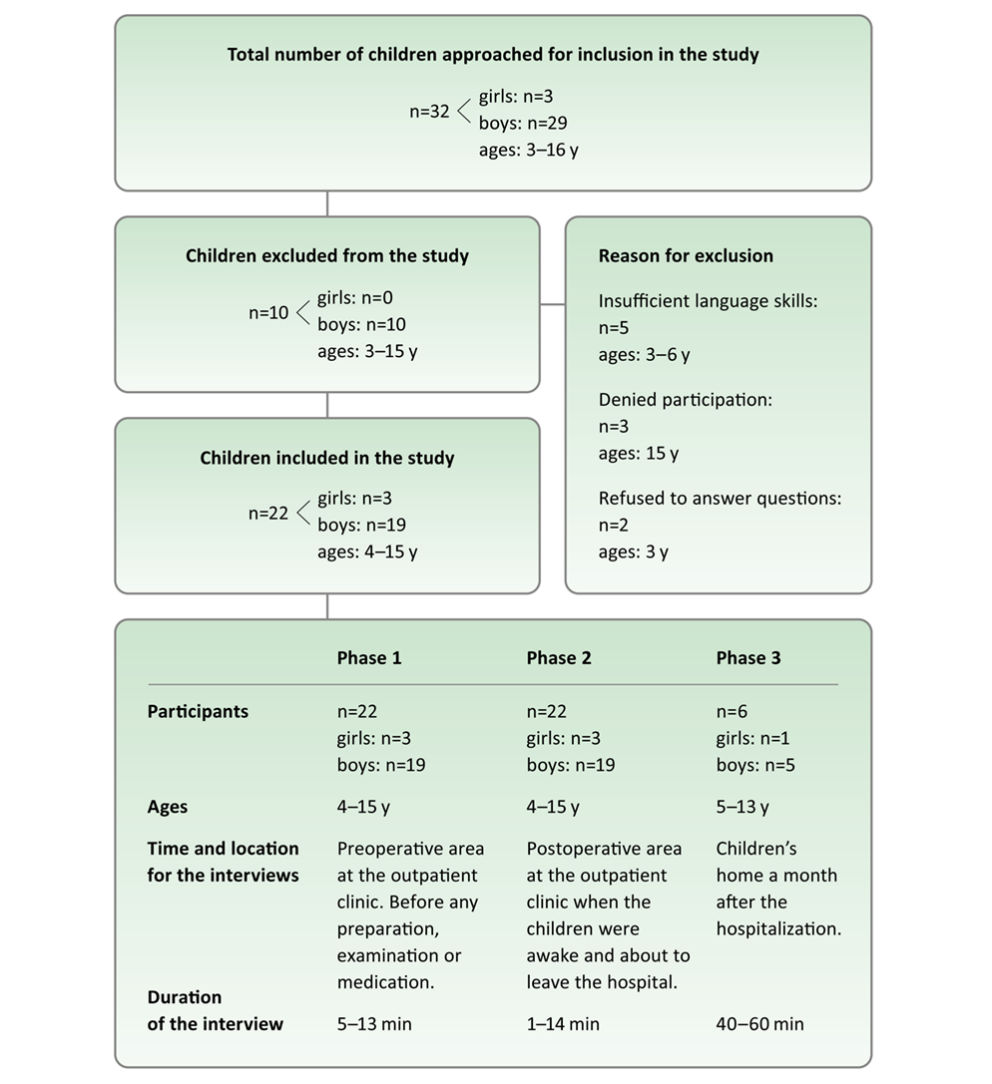
Overview of the participants and interviews conducted in the three-phase study.

The data in this part of the study (phase 3) relates to six children (ages 5 to 13 years) who participated in the follow-up study in their homes a month after hospitalization. The children were selected from the 22 originally interviewed (in phases 1 and 2) to represent a variation of ages and perioperative experiences (Figure 1).

To establish a relaxed atmosphere, the meeting in the child’s home began with play or an informal conversation about the child’s interests and daily activities. The interviews were performed, without any parent present, in a room chosen by the child. The interviews were conducted in a semistructured manner when children visited the website. The children maneuvered the website without guidance or influence from the interviewer. Field notes of the children’s actions were taken. The conversation was adapted to the individual child’s existing cognitive developmental stage.

To explore the children’s experiences and comprehension of perioperative processes in relation to their use of the Web-based preparation program, they were asked to show the interviewer around the website. Children were encouraged to explain the reason for the route chosen in navigating the website, describe the content and context, as well as their positive and negative experiences in relation to what they encountered on the website.

To encourage children to expand on their experiences, they were asked “If you were to tell a friend about hospitalization, anesthesia, and surgery, how would you describe it?” (for children aged 3-6 years), “If one of your friends was about to be operated on, how would you explain about anesthesia and surgery?” (for children aged 7-11 years), or “What was your experience of hospitalization, anesthesia, and surgery?” (for children aged 12 years and older) [41]. The interviews were recorded and transcribed verbatim. Nonverbal communication, such as silences, looks, laughter, posture, and gestures, were also transcribed because these may influence the underlying meaning of responses [42].

### Data Analysis

The data were analyzed based on a combination of the transcribed interviews and field notes, which were subjected to directed qualitative content analysis. To ensure trustworthiness [40,43], the analysis of the text was performed in several steps by a research team consisting of three members with extensive knowledge within the areas of pediatrics, anesthesia, medical education, preparation of children for medical procedures, and of designing Web-based information in medical context.

In the first stage of the analysis, the recordings were listened to and the transcripts were read several times by one of the authors (GL) to ensure familiarity with the data and to get a sense of the whole. In the second stage, those parts of the text that appeared to be related to the predetermined themes were highlighted. In the third stage, the predetermined concepts for the children’s learning processes on the website were analyzed related to the children’s expressions, experiences, and use of the website during the interviews. Data that could not be related primarily to the predetermined themes were identified and saved. The data were interpreted to elucidate additional dimensions of children’s experiences and usage of the website. The interpretations were compared with the predetermined themes and described as a new theme. To ensure confirmability, each theme was linked to the data by quotations from children. The research team continuously reviewed the data to ensure that the analysis accurately reflected the children’s statements and performance on the website and to validate the predetermined themes reconciling any differences using the theoretical framework.

#### Predetermined Themes

**Figure 2:**
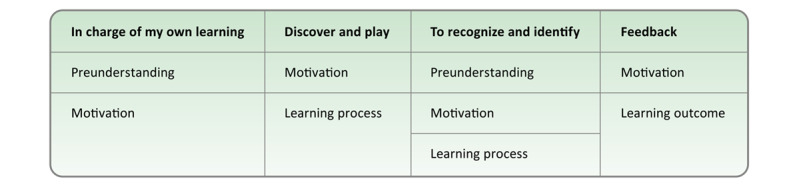
Predetermined themes related to children’s learning processes on the Anaesthesia-Web applied in the directed content analysis [18].

The predetermined themes applied in the directed content analysis were derived from the theoretical educational analysis of a Web-based preparation program, the Anaesthesia-Web [18]. The Anaesthesia-Web represents a comprehensive, interactive, age-differentiated multimedia Web-based portal that prepares and educates children and their families before pediatric perioperative care. The analysis related to central concepts for children’s learning processes on the Anaesthesia-Web resulted in the following themes: (1) in charge of my learning, (2) discover and play, (3) recognize and identify, and (4) getting feedback [18]. The correspondence between the concepts and the themes is presented in Figure 2.

#### Theme 1: In Charge of My Learning

The diversity and design of the website offer the visitors individual choice of how to enter and use the program. Children are in control and can use the website based on their varied backgrounds, abilities, and what they think is interesting and meaningful. This is important for motivation and each child’s individual preunderstanding.

#### Theme 2: Discover and Play

The content on the website is mediated by playful interactive elements to stimulate the children’s natural curiosity, creativity, knowledge seeking, and motivation—factors which are crucial to initiate and maintain the learning process. Through discovery and play, children can receive, process, and apply information cognitively, emotionally, and by active participation. Interactive parts of the website enable children to explore the hospital environment, prepare for upcoming events, and process any previous experiences.

By providing children with tools to help them understand and manage procedures, they may be able to relate simulated performances on the website with their perioperative expectations and experiences.

#### Theme 3: Recognize and Identify

On the website, children can recognize situations and feelings, interact with a diversity of characters, and find someone with whom to identify. They can also recognize and identify themselves as a person needing to learn and prepare before hospitalization. Recognition and identification are important factors to experience the visit as meaningful and to maintain the motivation to learn.

#### Theme 4: Getting Feedback

The website offers children opportunities to get feedback, which is crucial for keeping up the motivation to learn and is necessary to judge the learning achievements, confirm understanding, and the need for repetition. On the website, children get immediate feedback on their performance and progress with acknowledgment for achievements and performances. This increases motivation and concentration and stimulates the learning process.

## Results

### Overview

These results are from the last part of the three-phase study. Six children were selected from the 22 originally interviewed in phases 1 and 2, five boys (5-12 years) and one girl (13 years), and they were interviewed in their homes 1 month after hospitalization.

Children’s use of the website could be described through the predetermined educational themes on which the analysis was based. The analysis also clearly revealed a new theme: interaction with other children. This theme describes a dimension of recognition and feedback in which children’s interactions with other visitors on the notice boards came to the fore as important.

### Theme 1: In Charge of My Learning

Children’s entrance into the multimedia on the Anaesthesia-Web was based on individual choices characterized by a nonsystematic search to get an overview of the structure and content. Without instructions, the children surfed around the website. Independent of routes and choices taken on the website, they finally made individual choices and returned to parts they wanted to explore further. Characteristic of the children’s visits was an expressed desire to understand and cope with experiences from the hospitalization. The timeframe in which children stayed at the respective sections of the website differed depending on interest and experience. Parts of the website that caught the children’s interest were characterized by both long and recurring visits, triggering them to challenge their experienced fear, find answers to questions, and test their existing knowledge: “I want to go back to the film showing the bowels from the inside. Maybe I can find out why I have been in such pain when I understand how it looks inside me” (boy, 10 years). It was also obvious that the children choose different approaches for understanding the same problem: “First I learned about the intravenous needle by watching the film but since I want to find out more I also want to read about it and try doing it myself” (boy, 12 years).

In their individual choices of how to receive the information, the children used the open access of the website: “I am actually 12 years old but I think 13+ is best for me since I have been hospitalized before” (boy, 12 years). The children’s interest was captured by colors, sounds, and movement. The interactive parts of the website were described as fun, curious, and triggered the children’s motivation for exploration: “I am sure you can learn a lot on the reading part, but the interactive parts seem more fun since you can do things yourself there” (boy, 9 years). The children’s choices also reflected how their actual cognitive stage was affected by the situation: “Normally, I remember and learn best by reading, but in this special situation I think it’s better for me to receive information aimed for younger children” (girl, 13 years).

The children’s choices on the website reflected their desire to take command of decisions and actions. In the interactive operating room, the children recognized and discussed the frightening environment, furnishing, and technical equipment: “I wish I had my own operating room where I could decide and do what I want” (boy, 10 years). Based on individual choices and desires, they created their own operating room and suggested arrangements on how to decrease fears and make these surroundings suit children better. In the role play, the children took command over the selection of characters and roles: “I want to operate on the doctor. If he gets scared and starts to cry, I will just continue and say: you have to lie still, we have to do this, and this is not so dangerous?” (boy 5 years). The children’s choices of characters for the patient’s roles were similar to themselves in regard to age, diagnosis, procedures, and surgical interventions: “I want to play with a child with problems and pain from the same part of the body as mine” (boy, 9 years). The nurses and doctors for the role play were selected based on personalities “I think it is important that all nurses are happy and friendly” (boy, 5 years) and capabilities “Because there is a risk that older people may be a bit shaky in the hands, it is safest to choose a young surgeon” (boy, 10 years).

As the children were interacting on the website, they discussed and confirmed their own positive and negative experiences and proposed changes for the perioperative processes. Based on their own preunderstanding and the available content on the website, the children also expressed how and what parts of the website could be resources for other children’s preparation for perioperative processes: “The Anaesthesia-Web resembles the reality and you can decide yourself what to do. All children should visit the website to decrease their fears before admission to the hospital” (boy 12 years).

### Theme 2: Discover and Play

The interactive parts of the website were popular and the most visited by all children. With the interactive elements, the children explored the hospital environment and perioperative processes and compared them to what they had understood and remembered from their own visit. With great enthusiasm, they discovered the playful interactive parts simultaneously as they described what they were doing and the reason for their actions. It was also obvious that the playful and interactive parts of the website were important for the children’s processing of events and a useful tool in their description and explanation to others about procedures. The children created personal patches and bandages and decorated and furnished the interactive operating room to their own desire and taste. Based on their impressions of the hospital environment as cold, rigid, and with boring furnishing, they created their “dream hospital” (boy, 9 years). They painted the walls and floors in the operating room with strong and bright colors because “it would be less frightening for children going in to the operating room if the surroundings were more colorful” (girl, 13 years), and with a camouflaged pattern because “in films there are always blood on the walls and floors in the operating room” (boy, 10 years). When the children played, they chose a comfortable and soft operating table and a less hospital-like operating lamp, which both could be maneuvered by them.

The children’s play was permeated by practical applications of their own experiences of undergoing treatments and procedures. They drew many and long marks on the patients to be operated on. They also argued the need for several intravenous accesses, which would need innumerable attempts for insertion. They confirmed the need for procedures and convinced themselves it would be less painful and frightening than expected. During the practical exercises, they identified events and put words on concepts and objects: “It’s so much easier to understand how things work when you’ve tested yourself” (boy, 9 years).

When the children were playing on the website, their need to understand the procedures surrounding the insertion and use of the intravenous needle was prominent: “The use of the local anesthetic cream will make it less painful and, look carefully, I remove the needle and throw it away, it is only a small plastic hose remaining which does not hurt at all” (boy, 11 years).

When playing with the anesthesia machine and the monitors, the children related the anatomical and physiological conditions to the measurement values and suggested a possible need for action: “You’d better take deep breaths to get more oxygen in the lungs when the saturation is low” (boy, 11 years). The children’s curiosity about body functions and how body parts and organs are related to one another were also prominent when they were playing games on the website. Without any signs of being bored, the children used puzzles and games on the website to try to find out about the body, illness, and treatments.

### Theme 3: Recognize and Identify

After the children’s initial tour around the website, their individual choices focused in on what they could identify and recognize from their own hospitalization. The children recognized the hospital environment, technical equipment, and procedures: “The website is very similar to the hospital and everything that happens there. Yes, it is almost as the reality” (boy, 9 years). Confirmed was also the website’s reliability: “The website tells you how it will be at the hospital. You will get to know if it will be as painful as expected and be assured that everything will work out well” (boy, 12 years). It was also obvious that orders of procedures were noticed and that these were important for the children’s identification and recognition: “Everything that happened to the children on the website also happened to me, exactly in the same order” (girl, 13 years). Adjustments to processes were another factor children recognized and expressed as important: “My mother was also allowed to be with me when I was anesthetized, but she had to wait for me outside during the operation, that’s the rules” (boy, 9 years).

The children identified and recognized preoperative orders “The clown is not allowed to eat any breakfast on the day of surgery, but maybe she can drink some clear liquid” (boy, 9 years) and frightening and painful procedures “It will hurt very, very much for the clown to get an intravenous access, I can see how scared she is” (boy, 5 years). The children identified their own symptoms, discussed possible investigations and treatments, and compared these to the medical conditions of the characters on the website: “I can exactly recognize the sour taste coming in the throat from the stomach. Maybe there is a need of an examination and some medication for this child” (boy, 10 years). They also identified their own medical condition and experiences in relation to the health care providers: “Think if the doctor had a problem with peeing, had to get a urine catheter and maybe operated on” (boy, 5 years). With help of the website’s key feature, Hilding Vilding, the children were able to recognize and identify fears and worries simultaneously as reflecting their experiences to someone always doing worse: “Hilding Vilding is doing what you are not allowed to at the hospital and he is afraid of needles as me” (boy, 5 years).

### Theme 4: Getting Feedback

The different opportunities for the children to receive feedback on the website were expressed as the most important to confirm their experiences and understanding, and expand and judge their learning. By receiving feedback, the children were given opportunities to apply ideas, correct and learn from errors, improve performance, and achieve goals. The children appreciated the help from the website’s main feature, Doctor Safeweb, to receive feedback on quizzes, games, and frequently asked questions, but they also sometimes found his personality disturbing: “Lucky you can choose to turn off his voice in the background when he gets too nagging” (boy, 10 years). They also highlighted the importance of having different levels of the challenges and frequent opportunities to apply their ideas: “It is fun when I can test how much I understand and have learned in different ways on the website” (boy, 11 years). When the children were testing their knowledge in games and quizzes, they were triggered and motivated to continue when they received immediate answers, and their progress was visible in any way. The children showed impatience and continued to other parts of the website when the content and layout did not catch their attention or when the feedback was slow: “I like the short films when you quickly get to know how it goes and do not have to wait for the answer” (boy, 10 years).

### Theme 5: Interaction With Other Children

The children’s identification with others in the same situation was prominent when they were visiting the notice board. Children appreciated being able to chat, ask questions, and receive answers from others: “It feels good to know how and what others felt, thought, and wondered” (boy, 11 years). They confirmed that many of the children writing on the notice board shared their own experience of being uninformed, having many questions, and being afraid of what to expect at the hospital. They highlighted the importance for these children to receive information before their admission because it had been crucial for their own preparation. When the children were reading other children’s notes, they were concerned that many questions remained unanswered: “It must be such a terrible feeling to be afraid and not having their fears addressed” (girl, 13 years). Prominent in the children’s identification with other visitors was also their recognition of how previous medical experience did not protect them from being afraid. On the contrary, it often meant the opposite: “I know exactly how they feel. To know what to expect also makes me even more nervous” (boy, 12 years).

The children used all opportunities for feedback on the website: they not only received feedback but they were also giving feedback on the notice board to other children’s questions and concerns. Advice on how best to relate to events and procedures was frequently exchanged with other visitors. Based on identification and recognition of their own experienced fear, the children wrote advice in the notes as they verbally expressed how to best prepare for and handle events to reduce stress and anxiety during the hospitalization: “It is normal and legitimate to be afraid for being hospitalized, but believe me you don’t have to be frightened, they have total control” (boy, 11 years). The children also identified with and replied to commonly asked questions from children on the notice board function of the website. Of special concern was the fear of the intravenous access “The local anesthetic cream works very well so it will not be painful at all to get the intravenous access” (boy, 12 years) and for anesthesia and painful procedures “You will fall asleep very fast and not experience any pain at all during the surgery since the nerves will be anesthetized by the sleeping medication” (girl, 13 years). The children also identified and replied to questions associated with the postoperative procedures: “It is normal to have pain after surgery, but you don’t have to be afraid. Just relax and take some painkillers, it will help” (boy, 11 years).

## Discussion

The goal of this study was to provide theoretical and empirical knowledge as a basis for the design and development of websites for children’s learning in pediatric care. Such websites should not just provide information, but should also stimulate learning. This analysis shows how this could be achieved.

The themes derived from a previous theoretical analysis of a website [18] were recognized in the children’s use of the website in this study. In particular, the themes “in charge of my learning” and “discover and play” corresponded well with the theoretical educational assumptions of the design and the children’s use and behavior. Correspondence was also found with the predetermined themes “recognize and identify” and “getting feedback.” In addition to these themes, aspects which appeared as significant for children’s needs and interest in processing experiences and information related to the perioperative processes came to the fore. To capture these aspects of learning and to highlight the importance of children’s needs for interaction with others facing similar experiences, the additional theme “interaction with other children” highlights an important part for consideration in the development of websites in pediatric care.

The interpretations of the children’s statements and actions when using the website revealed how they took advantage of all available opportunities in making their own choices to manage their experiences from the hospitalization. This is consistent with results from previous studies about children’s engagement on websites with open content and possibilities for choices [33]. The different possibilities for them to be in charge of their own learning were shown as important driving forces both to start and proceed with their search for learning. To consider assumptions about the significance of preunderstanding and motivation in children’s learning processes thus seem to be important factors in the design of websites in pediatric care. Preunderstanding is significant for learning as it constitutes the base for interpretations, thoughts, and understanding of new experiences [19,21]. The development of Web-based learning opportunities for children in pediatric care thus should be designed including prerequisites for individual interactions by using complementary multimedia and sensorial features to offer children a choice of interfaces. In addition, the child should be offered control to stimulate triggers for their learning activities. This can be achieved by offering free access to the diversity of multimedia to choose when, how, and what information to receive [18].

A large proportion of the time the children spent on the website was related to play and discovery both to confirm and process experiences and to stimulate their curiosity to find out and learn new things related to their body, perioperative processes, and the hospital environment. By playing the different roles of the professionals, in a virtual reality, children were exposed to and learned about the setting itself as they played and interacted. As the children were playing, they verbally expressed and explained what they were doing and the reasons for their actions.

Creating possibilities for play and interactive participation is known to be of key importance in the development of Web-based learning opportunities for children [18,33,35]. This was confirmed in this study where it was obvious that children virtually can prepare for and process experiences by practicing skills and procedures. With the help of Web-based technology, children can be exposed to and learn about the surroundings and procedures that would otherwise be inaccessible for them [32].

Opportunities for choices and creativity are known to be important for children’s learning [33]. Thus, we would highlight the importance of combining possibilities for children to discover and play with possibilities to be in charge of their learning. To stimulate and maintain motivation through children’s curiosity and knowledge seeking, the content and design of websites in pediatric care should be developed to be filled with fun and excitement to explore [33]. With the use of creative tools involving all senses, children can be given opportunities for interactive imaginative expressions to increase interest and engagement as a base for their learning processes [21].

Children’s need for identification and recognition were prominent when they were using the website. They interacted with the diversity of characters and perioperative procedures on the website and displayed ability and a need to express, engage in, and compare to their own experiences. It seems these interactions helped them challenge their fears and legitimize their own worries and need for help as well as to keep up their motivation to understand and learn more. To optimize children’s prerequisites for identification and recognition, they should have the ability on websites in pediatric care to interact both with common procedures and with a diversity of characters. It is also important to include opportunities for children to recognize the emotional aspects they may experience in hospital [14,15].

The website’s different opportunities for feedback on the processes and performances emerged as central to help the children understand what was going to happen and to experience safety and confidence. The children used the available chances to correct, learn from errors, and improve performances. By using different levels of challenges and frequent opportunities to apply ideas, they strived to receive feedback on actions taken on the website. Therefore, feedback received on the website constituted an important factor in confirming the children’s experiences and learning outcomes and in keeping up their focus and motivation [29,30,34]. Yet, the feedback the children received on the website was mainly related to their understanding of the perioperative processes, and not individual feedback on their experience and emotions. The additional theme connected to the children’s need for identification, recognition, and feedback that appeared in the analysis is connected to this need for individual feedback. The notice board, which gave the children access to other children’s experiences, resulted in deep concerns and engagement. The children’s usage of the website to not only receive feedback on their own experiences but to also interact and give feedback on other visitor’s questions and concerns emerged as very important. It was obvious that the children had a need for access to a platform where they could identify, interact, and learn from other children’s perspectives. In turn, that led to increased motivation to reflect on and learn about their own situation.

The results from this study showed that children’s use of and experiences with a website such as this can be explained through the educational themes defined in a previous theoretical analysis [18]. The study also reveals new aspects that need to be taken into consideration and further developed when designing Web-based learning opportunities in pediatric care. The children used the different opportunities for receiving feedback on the website, but it was obvious that this area constitutes a vulnerable and challenging part in the configuration of websites in pediatric care. Careful consideration has to be taken when the learning aims for the child are to be prepared for situations filled with unfamiliar and emotional components. In addition, this learning process mostly takes place at home where the possibility of correction of misunderstandings and inaccuracies cannot be taken for granted. The results of the children’s learning outcome will not be obvious until the day of admission at the hospital.

During the children’s performances and use of the website, it was evident that their processing of experiences and gathering of information included both interactive actions and verbal reasoning about feelings and experiences. The opportunity for children to reflect on their experiences to an adult constituted an important component in their processing of experiences and continuation of learning processes on the website. In this case, the researcher represented the adult connection. Without controlling or affecting their children’s ability to be in charge of their own learning, parents should visit the website together with their child sometimes. In addition, we would suggest future development of websites within pediatric care to consider possibilities for children to chat and receive feedback on questions and concerns direct from medical professionals.

This analysis of children’s use and experiences of a Web-based perioperative preparation program emphasizes the importance of including the central learning concepts of preunderstanding, motivation, learning processes, and learning outcome in the development and design of websites in pediatric care [18]. Creating opportunities for Web-based communication with others facing similar experiences and possibilities for receiving feedback from adults constitute additional inclusive factors for consideration.

Although this study was aimed to be carefully prepared and accomplished, some methodological limitations need to be highlighted and discussed. The choice of learning theories and the assumptions about learning they mirror will influence the analysis and the result. The theoretical educational framework directs the attention of the researchers in the analysis process and also permeates the interpretations. To ensure trustworthiness, the theoretical educational framework are described in detail (see Multimedia Appendix 1) and applied systematically [43] by an experienced multidisciplinary group. The research process and context have been thoroughly described to enable researchers and other readers to transfer the results to other contexts. One limitation in this study was that the children were recruited from the same health care setting, were relatively healthy, and undergoing routine surgeries. Another limitation was the imbalance of gender, which is explained by the type of surgeries performed during the week of data collection. Although the number of participants was small, the configuration of the study provided continuity of children’s experiences which, in combination with the qualitative approach, provided deep insights into the children’s perspectives.

This analysis of children’s use of and experiences with a Web-based perioperative preparation program shows the significance of including a theoretical educational framework of children’s learning in the development and design of websites in pediatric care. In this study, the central learning concepts of preunderstanding, motivation, learning processes, and learning outcome represented a useful general educational level of inquiring into children’s learning using a website. The most important characteristics supporting children’s learning were found to be in charge of my learning, discover and play, recognize and identify, getting feedback, and to be in interaction with other children. The last theme emphasizes that creating opportunities for Web-based communication with others facing similar experiences and possibilities for receiving feedback from adults have to be carefully considered in the future development of websites preparing children for perioperative care.

## Appendix

Multimedia Appendix 1 Learning concepts.
